# Complete Extrusion of Talar Body Associated With Ipsilateral Floating Knee

**DOI:** 10.7759/cureus.10346

**Published:** 2020-09-09

**Authors:** Mohamad K Moussa, Ryan Bou Raad, Ismat Ghanem, Oussama Mansour

**Affiliations:** 1 Orthopedic Surgery, Lebanese University Faculty of Medical Sciences, Beirut, LBN; 2 Pediatric Orthopedic Surgery, Hôtel Dieu de France Hospital, Beirut, LBN; 3 Orthopedics and Traumatology, Al-Zahraa Hospital University Medical Center, Beirut, LBN

**Keywords:** talus extrusion, staged fusion, cement spacer, arthrodesis, pantalar dislocation, floating knee

## Abstract

Talar injuries represent serious medico-surgical conditions because of the involvement of the talus in multiple articulations, such as the subtalar, the transverse talar, and the ankle joint complex. Its complete detachment from the surrounding ligaments and bone is known as talar extrusion, a very rare injury with a complicated treatment course.

We report a case of a 43-year-old female patient presenting with a non-retrieved complete left talar body extrusion associated with a floating knee, manifested by left tibial shaft fracture and left supracondylar femoral fracture. The patient was treated with open reduction and internal fixation for the floating knee and a manually shaped talar cement spacer with staged tibiocalcaneal arthrodesis for the ankle. The patient was monitored over a six-year period.

This case is reported for the extreme rarity of lost open talar body extrusion, and its problematic treatment in the absence of clear guidelines, especially with the presence of multiple concomitant ipsilateral fractures of the limb, such as floating knee, as in this case.

## Introduction

Complete talar extrusion, often caused by high-energy trauma, is a rare injury that is seldom mentioned in literature [1]. It is most commonly associated with an anterolateral ankle wound, as well as other injuries such as neck fractures, fracture dislocations, and severe soft tissue injury. The mechanism of injury is a combination of tibiotalar plantar flexion with excessive supination or pronation.

The rarity of this case makes the establishment of a treatment protocol a difficult task to tackle, and to our knowledge, no consensus exists on a treatment algorithm. Several treatment modalities have been proposed with variable results, ranging from reimplantation, arthrodesis, and talectomy [2-4]. Continuously emerging therapeutic options often leave the choice to the surgeon to determine the optimal strategy based on the presenting case. Ankle disabling complications may arise during the treatment course, such as infection, avascular necrosis, and osteoarthritis.

In this article, we present an uncommon case of open talar neck fracture dislocation and body extrusion in a 43-year-old woman, where the talar body was lost on the scene, associated with a floating knee from closed tibial shaft fracture and closed supracondylar fracture.

## Case presentation

**Figure 1:**
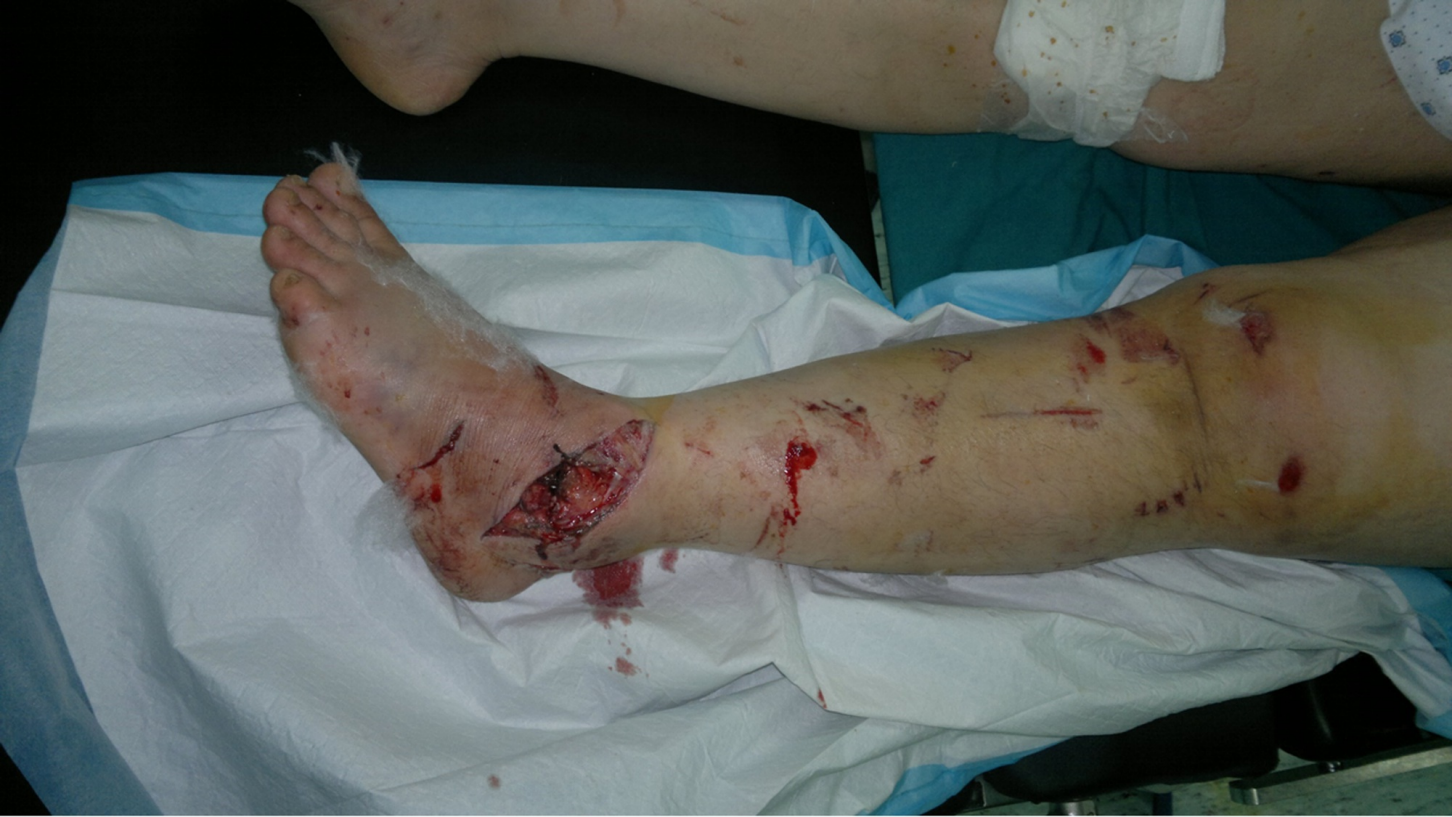
Inspection of the left lower limb showing a wound on the anterolateral aspect of the ankle and an internally rotated foot.

This is a case of a 43-year-old female patient presenting to the emergency department after a motor vehicle accident. Upon physical examination, the patient had a deformed left lower extremity, hemarthrosis of the left knee, a wound on the anterolateral aspect of the ankle, and an internally rotated left foot (Figure 1). Neurovascular exam was normal.

The wound was heavily irrigated and closed primarily. The patient was given intravenous antibiotics and tetanus prophylaxis.

**Figure 2:**
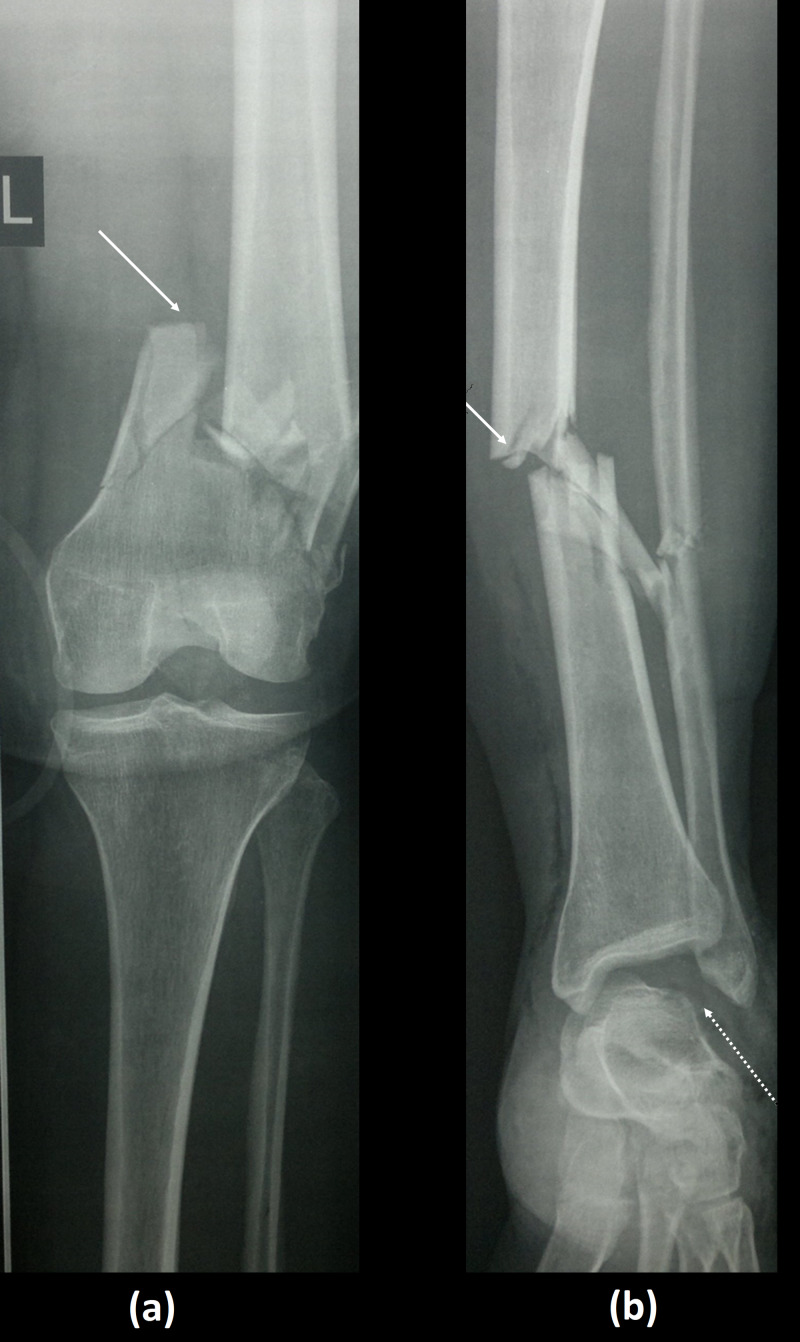
(a) AP radiograph of the left knee showing left extraarticular distal femur fracture. (b) AP radiograph of the left leg showing tibial shaft fracture (solid white arrow), and a complete absence of the talar body (dotted arrow). AP, anteroposterior

Initial radiographs of the left lower limb showed a left-sided extra-articular distal femoral fracture, associated with a tibial shaft fracture, and an ipsilateral complete talar extrusion (Figure 2).

A diagnosis of floating knee associated with talar neck fracture with complete extrusion of the body leaving the head in situ, and the family was asked to extensively search for the lost talar bone at the scene of the accident. After two hours of searching, the talus was not retrieved.

**Figure 3:**
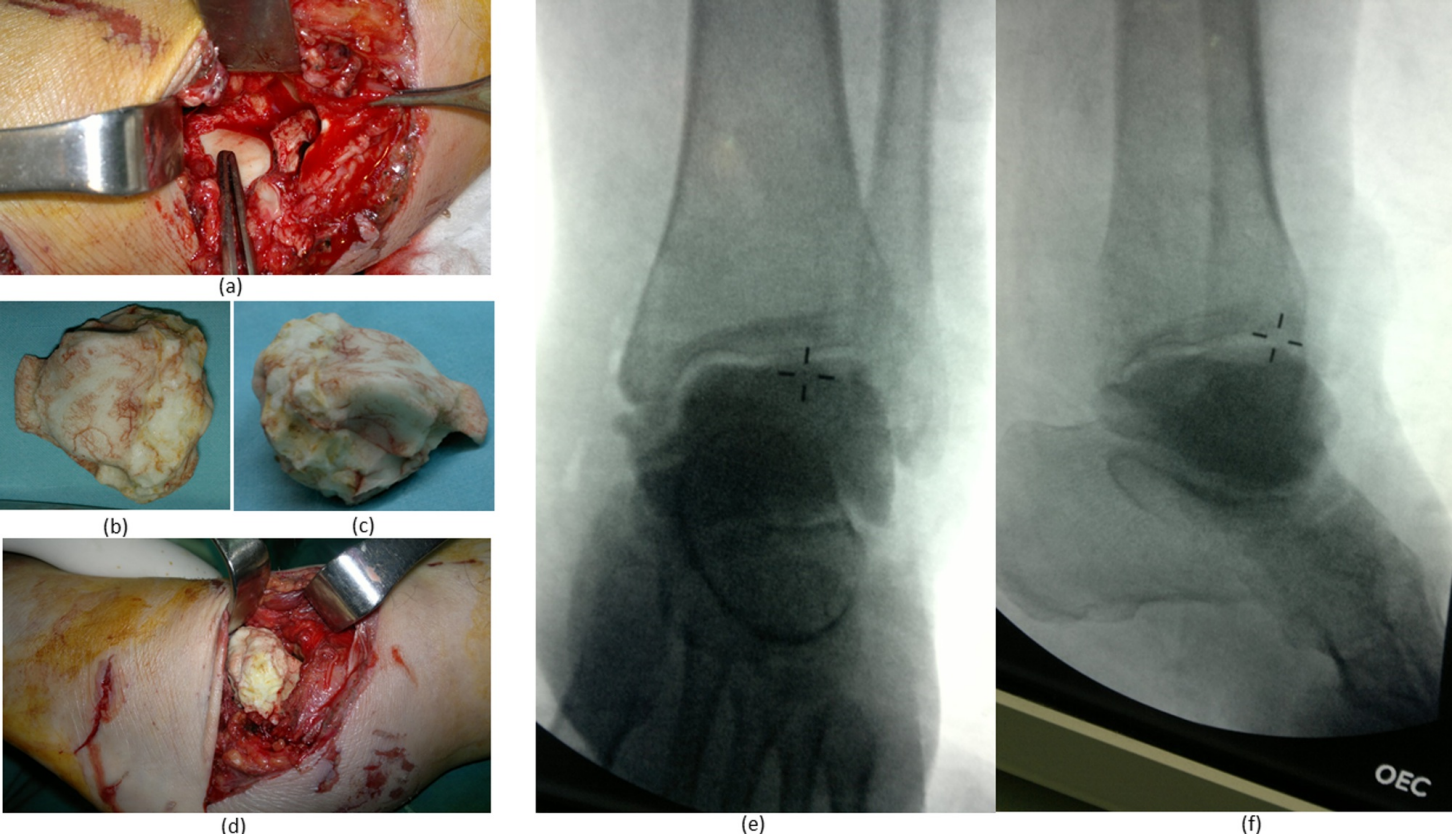
Different aspects of the surgical intervention at the ankle level: (a) exposure of the tibial mortise after copious irrigation and debridement, (b) superior view of the handmade talar-shaped cement spacer, (c) side view of the handmade talar-shaped cement spacer, (d) implantation of the cement spacer, (e and f) fluoroscopic guidance to ensure adequate positioning of the spacer.

The patient was informed about the remaining treatment options, and the decision was made to undergo a two-stage treatment. The patient was then brought to the operating room. At the ankle site, the wound was opened, and debridement was carried out with copious irrigation. A talar-shaped cement spacer, impregnated with vancomycin and tobramycin, was manually crafted and then accurately implanted in the mortise under intraoperative fluoroscopy (Figure 3). The wound was irrigated again, and the skin was closed.

**Figure 4:**
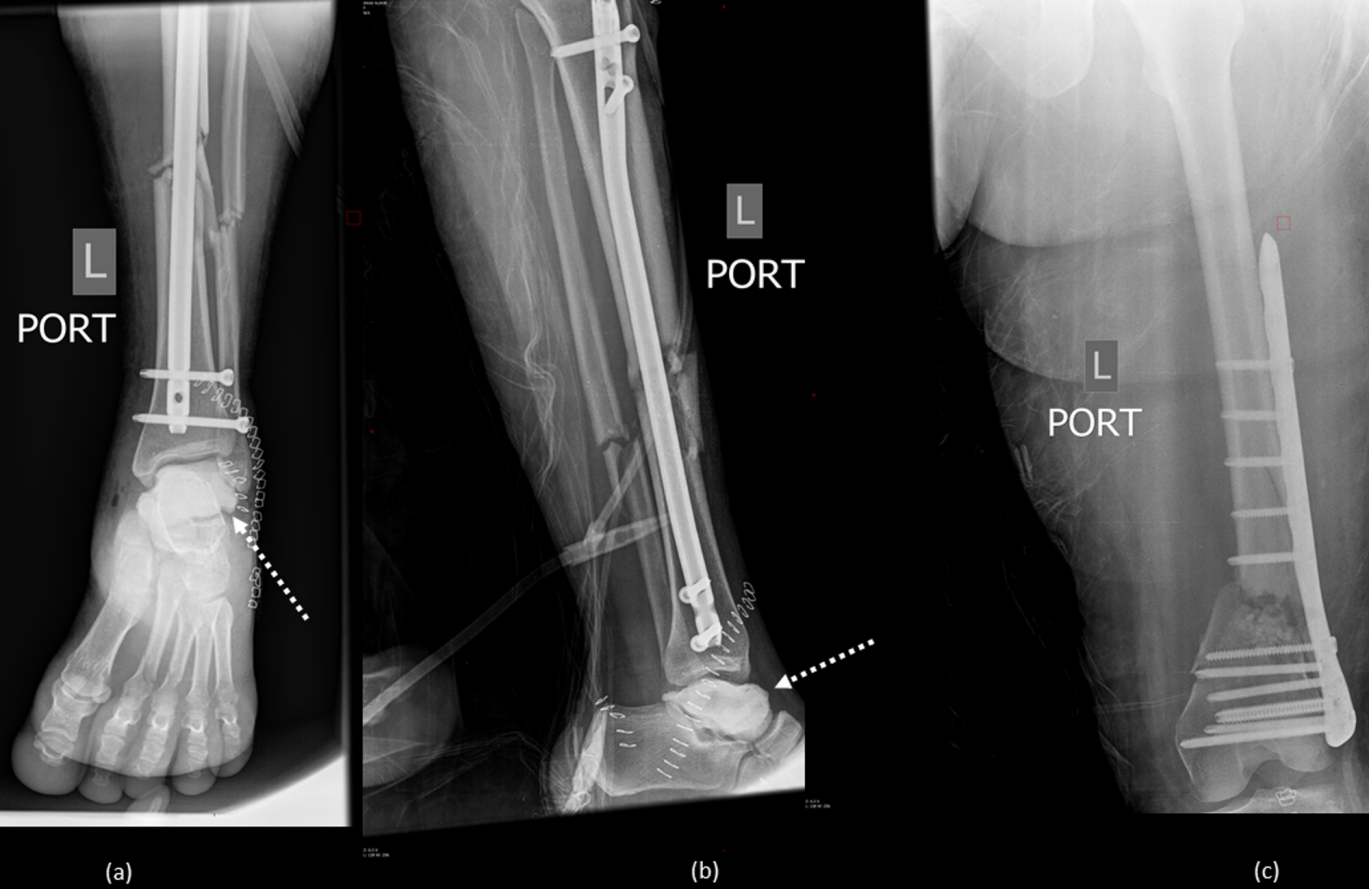
(a) AP view of the left ankle showing osteosynthesis of the tibial shaft, with intramedullary nail and talar-shaped cement spacer in place (dotted arrow). (b) Lateral view of the leg showing osteosynthesis of the tibial shaft, with intramedullary nail and talar-shaped cement spacer in place. (c) AP view of the femur showing osteosynthesis of the supracondylar fracture by plate and screws. AP, anteroposterior

The floating knee was addressed with open reduction internal fixation with plate and screws for the femur, and intramedullary nailing for the tibia. Postoperative radiographs are shown in Figure 4.

Postoperatively, the patient was doing well at first. However, at the ankle level, she was experiencing a mild-to-moderate mechanical pain responsive to painkillers. The pain became severe when partial weight-bearing was allowed after 16 weeks. It was thus postponed.

**Figure 5:**
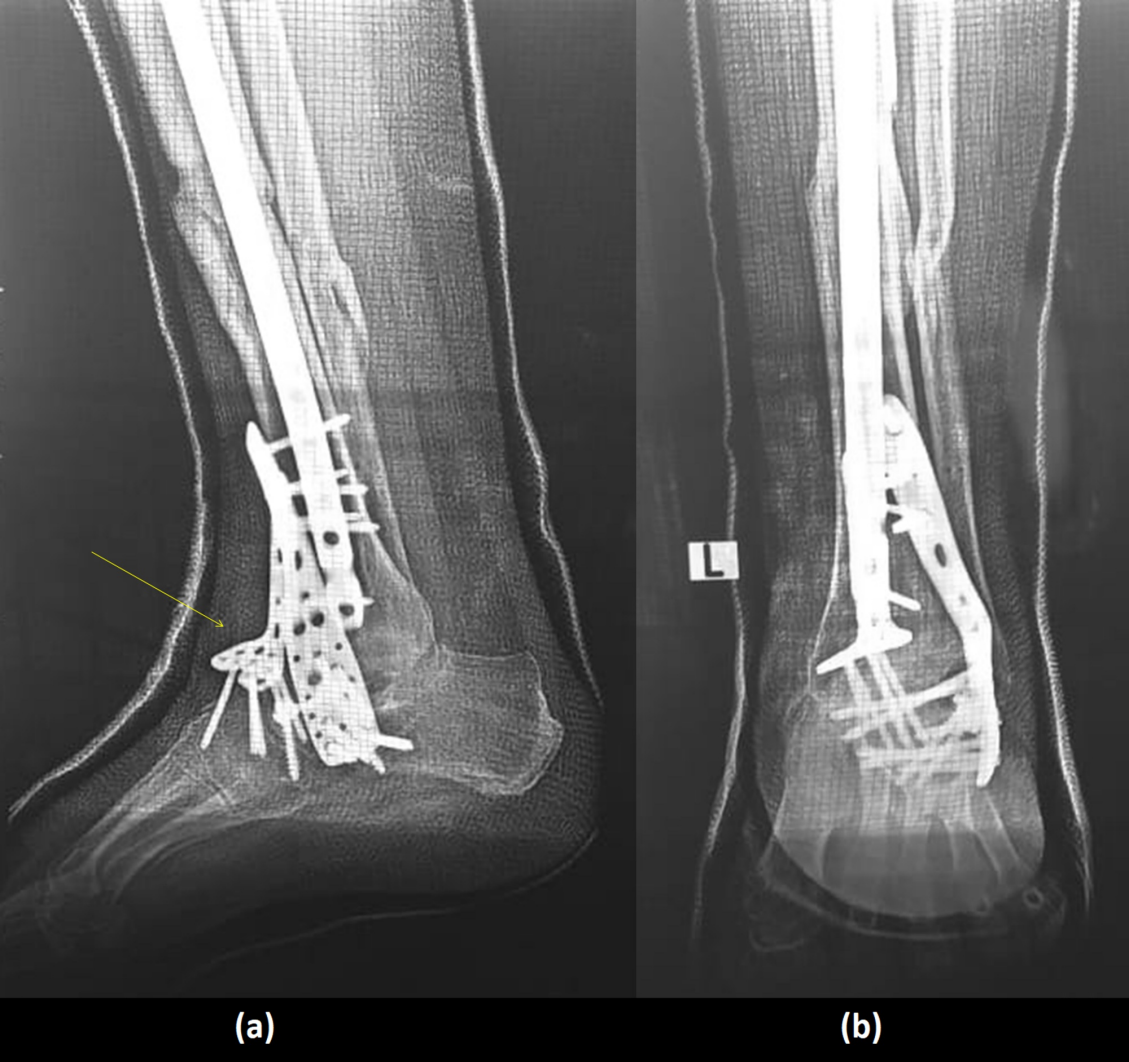
Radiographs post-tibiocalcaneal arthrodesis: (a) anteroposterior view and (b) lateral view.

Twelve months postoperatively, the pain became refractory to painkillers. Therefore, a second-stage reoperation with the removal of spacer and ankle tibiocalcaneal arthrodesis with retrograde ankle fusion nail was planned. Unfortunately, the distal locking screw of the previously inserted tibial nail was broken during attempted removal, so we shifted to fusion using two plates and screws, augmented by a synthetic bone graft. Postoperatively, the patient had significant improvement with complete resolution of the ankle symptoms, and three months postarthrodesis, she had complete radiological union. Postoperative radiographs are shown in Figure 5.

**Figure 6:**
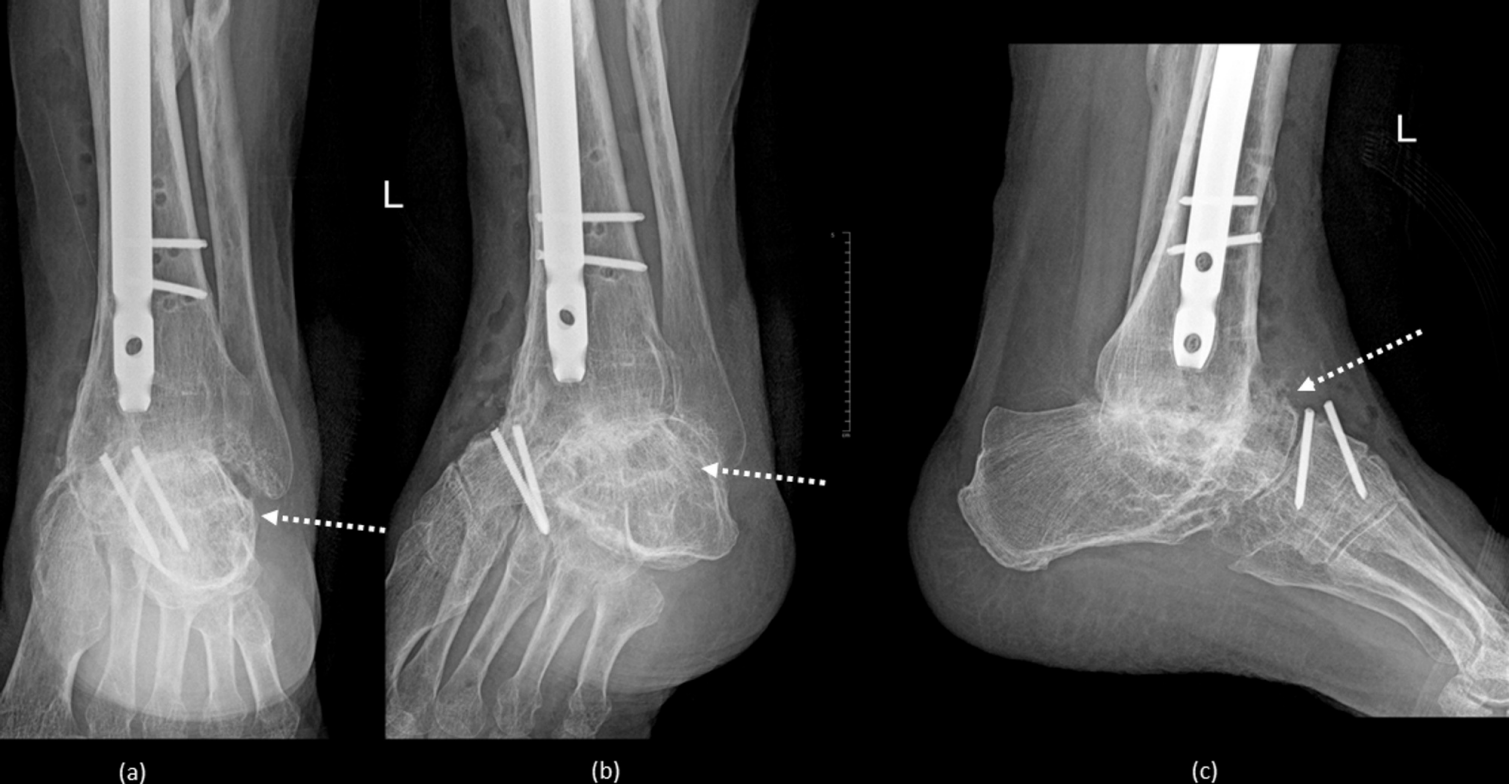
Radiographs post-removal of material and complete fusion of the tibiocalcaneal arthrodesis: (a) anteroposterior view, (b) oblique view, and (c) lateral view.

Retained orthopedic devices were removed at 18 months of arthrodesis, and the final result is shown in Figure 6.

## Discussion

Talar extrusion is extremely rare. While its true incidence is unknown, it is estimated at approximately 0.06% of all dislocations and 2% of all talar injuries [5]. Likewise, complete talar body extrusion is seldom mentioned in literature [2].

Therefore, due to the lack of sufficient data, there is no consensus on the best treatment protocol. Furthermore, the treatment course may be complicated by several limb-threatening conditions, such as infection, avascular necrosis, length discrepancy, and osteoarthritis.

In their series of 19 patients, of whom 14 had open fractures and were treated with various techniques, Boden et al. recently reported an 88% rate of osteonecrosis, a 44% rate of osteoarthritis, and no deep infections [6].

However, Marsh et al., in 1995, associated talar extrusion with 38% of infection rate, which led to their recommendation of primary talectomy to avoid this severe complication [7].

Various therapeutic options have been suggested in the literature, but comparison studies are lacking to determine the superiority of one strategy over others.

While in the past, talectomy with tibialcalcaneal arthrodesis was the mainstay of treatment [8], many authors recently reported reimplantation as a viable option, with successful results. This technique is favored because it preserves joint height and ankle biomechanics [2,9].

In their 2011 report, Vaienti et al. even yielded positive results with delayed reimplantation. The total extrusion was treated initially with cement spacer and distally based sural fasciocutaneous flap, followed by delayed reimplantation and arthrodesis after confirming the negative cultures of the original talus. They suggested that the flap might have promoted revascularization, which ultimately avoided the avascular necrosis of the reimplanted talus [4].

Likewise, a two-staged approach with talar-shaped cement spacer, followed by femoral head allograft, was reported with favorable results. This technique also avoids the risks of osteonecrosis and infection associated with re-implantation, while maintaining limb length [3].

Regardless of the treatment choice, when facing an open pantalar dislocation, commonly accompanied by an anterolateral wound of the ankle, early surgical debridement is the most important initial step [2-9]. The use of an external fixator to stabilize the joint while waiting for definitive treatment could also decrease wound complications and allow proper wound care [3,4].

After aggressive surgical exploration and debridement, the surgeon must make the decision based on several factors, such as the availability of the native talus, its condition and degree of contamination or bone loss if available, the condition of the surrounding soft tissues, and patient preference.

The inability to retrieve the extruded talus, as in our case, eliminates reimplantation as an option. Conversely, the presence of ipsilateral floating knee, in addition to the dirty ankle wound, makes primary arthrodesis very difficult and challenging, with a high risk of complications such as infection.

These factors render the approach to this extremely rare scenario very challenging technically, with regard to the filling of the resulting defect.

The technique of using a talar-shaped cement spacer and staged arthrodesis yielded favorable outcomes, with the patient ultimately regaining a functional daily life with full weight-bearing on the injured foot.

## Conclusions

Complete talar extrusion remains an extremely rare condition that challenges even the most skilled surgeons. Controversy remains over the best choice of treatment for such cases. The complications potentially arising from the treatment course could severely impact the patient. However, there is a multitude of plausible therapeutic options with favorable outcomes.

Talar extrusion with inability to retrieve the native talus is an even more unusual presentation. We present this case report due to the rarity of lost talar body extrusion along with associated floating knee. Ideally, after removal of the cement spacer for arthrodesis, a femoral head allograft would be the better technique to avoid limb length discrepancy. However, the unavailability of bone banks in our facilities compelled us to dismiss it. This case report shows good clinical outcomes, with our approach of using a talar-shaped cement spacer followed by arthrodesis, which affords the patient non-painful full weight-bearing, without the need for reoperation.
